# Ethics of Care in Nursing: Protocol for a Qualitative Systematic Review and Best-Fit Framework Synthesis

**DOI:** 10.2196/85172

**Published:** 2026-09-10

**Authors:** Wimonpan Sungsakul, Sungworn Ngudgratoke, Nalinee Na Nakorn, Anusorn Koedsri

**Affiliations:** 1 Educational Measurement and Evaluation Program School of Educational Studies Sukhothai Thammathirat Open University Nonthaburi Thailand; 2 Boromarajonani College of Nursing Sunpasitthiprasong Ubon Ratchathani Thailand; 3 Office of Registration, Records and Evaluation Sukhothai Thammathirat Open University Nonthaburi Thailand

**Keywords:** ethics of care, nursing, Tronto, Gilligan, systematic review, best-fit framework synthesis

## Abstract

**Background:**

Ethics of care lies at the heart of the nursing profession. Grounded in the work of Gilligan and Tronto, it emphasizes relationships and responsiveness to the needs of those receiving care. Although Tronto’s framework specifies 5 elements of care, no systematic synthesis has yet examined how each element is expressed through observable behavioral indicators in the nursing context.

**Objective:**

This review aims to identify, appraise, and synthesize the components and indicators of the ethics of care in the nursing profession, drawing on the frameworks of Gilligan and Tronto. It addresses 4 research questions concerning the components of the ethics of care, the indicators or behaviors that reflect each component, any indicators or aspects not yet captured by the existing frameworks, and how the components and indicators vary across contexts.

**Methods:**

This qualitative systematic review will use best-fit framework synthesis. Five electronic databases (CINAHL Complete, PubMed or MEDLINE, ScienceDirect, SpringerLink, and JSTOR) were searched, supplemented by backward citation searching of the reference lists of included studies, without date restriction and limited to English-language publications. The methodological quality of included studies will be appraised using the JBI tools to inform interpretation rather than as an exclusion criterion, and data will be synthesized in NVivo. The review will be reported in accordance with the PRISMA (Preferred Reporting Items for Systematic Reviews and Meta-Analyses) 2020 and PRISMA-S (Preferred Reporting Items for Systematic Reviews and Meta-Analyses literature search extension) guidelines.

**Results:**

This study received no external funding. The searches across the 5 databases were conducted in May 2026 and yielded 30,134 records; after the removal of 4855 duplicates, 25,279 records remained for screening. At the time of submission of this revised manuscript (May 2026), screening and data extraction were underway, and the review was anticipated to be completed by December 2026, with results expected to be published in 2027.

**Conclusions:**

The synthesized definitions and indicators will provide a foundation for developing assessment instruments and practice guidelines for the ethics of care in the nursing profession, as well as for advancing nursing education.

**Trial Registration:**

PROSPERO CRD420251032024; https://www.crd.york.ac.uk/PROSPERO/view/CRD420251032024

**International Registered Report Identifier (IRRID):**

DERR1-10.2196/85172

## Introduction

Nursing is fundamentally rooted in humane care, respect for human dignity, and attention to the individual needs of each patient [1]. Although times have changed and scientific and technological advances have emerged, the heart of nursing remains compassionate, humane care. Nursing care is distinct from caregiving in society more broadly; it encompasses medical treatment, interpersonal relationships, attentiveness, concern, and moral and ethical values, integrating the art of caring with knowledge from nursing science [2]. The profession is guided by ethical principles and standards that ensure quality care and uphold human dignity [3]. Nursing ethics is a core attribute of the profession, reflecting its values and professional identity, and it serves as a crucial guide that enables nurses to make complex ethical decisions in diverse and challenging situations [4,5].

Ethics in the nursing profession has often been discussed in the context of the ethics of justice, which emphasizes the equitable provision of care to all individuals regardless of race, religion, or social status. It involves treating every patient with equal respect and dignity and protecting patients’ rights. Rest [6] and Beauchamp and Childress [7] made significant contributions to the development of ethical frameworks for nursing grounded in the ethics of justice perspective. Key concepts include (1) moral sensitivity: the ability to recognize that a situation contains an ethical issue and to understand that one’s actions may affect others, (2) moral judgment: the capacity to analyze and determine which action is ethically right on the basis of principles and ethical reasoning, and (3) moral motivation: the prioritization of ethical values over other considerations, such as placing equitable patient care above personal gain. This perspective also encompasses moral courage, commitment, and ethical resilience—the strength and determination to act on what is right despite difficulty or external pressure.

**Figure 1:**
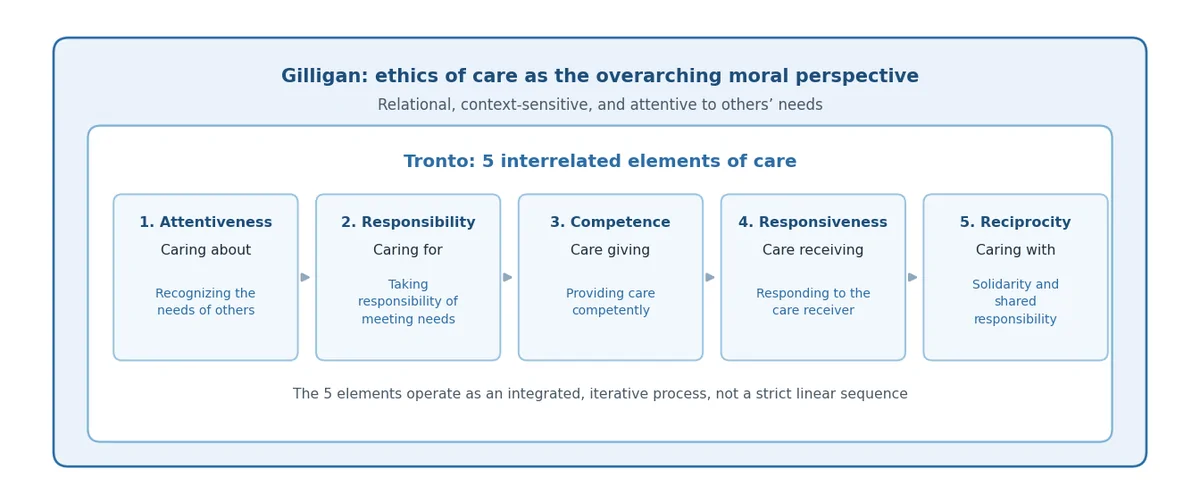
Conceptual framework guiding the review, integrating Gilligan’s [8] ethics of care as an overarching lens with Tronto’s [10] 5 elements of care (attentiveness, responsibility, competence, responsiveness, and reciprocity). Gilligan provides the overarching moral perspective; Tronto's 5 elements provide the operational structure. This review synthesizes the definitions and behavioral indicators of each component and maps the components (research question [RQ] 1) and observable indicators (RQ2) of the ethics of care in nursing practice and nursing education. These elements provide the a priori codes for the best-fit framework synthesis.

However, ethics in nursing also aligns with the theory of care ethics, or the ethics of care, proposed by Gilligan [8], which emphasizes caring action, the importance of personal relationships, and attentiveness to the specific needs and contexts of others. Tronto subsequently developed this perspective into a more concrete and practical form, proposing 4 phases of care in *Moral Boundaries: A Political Argument for an Ethic of Care* [9] and adding a fifth phase, “caring with,” in *Caring Democracy: Markets, Equality, and Justice* [10], yielding 5 elements: attentiveness, responsibility, competence, responsiveness, and reciprocity. These elements are highly congruent with the nature of nursing care. This review therefore adopts Gilligan’s [8] ethics of care as an overarching lens and Tronto’s [10] 5 elements as an operational structure, as illustrated in Figure 1. The concepts of care and justice are interconnected and mutually reinforcing in the delivery of effective nursing care [11-13].

In practice, nurses frequently encounter situations that give rise to moral distress (knowing the right course of action yet being constrained from pursuing it), as well as ethical dilemmas, in which 2 or more ethically defensible options exist without a clear resolution [14]. A clear understanding of what the ethics of care means, which components constitute it, and how it is expressed through behavior is therefore foundational to helping nurses navigate such situations.

Although the ethics of care is widely discussed in nursing, systematic reviews on the topic remain limited and vary considerably in their approaches. Selvakumar and Kenny [15] conducted a scoping review of empirical research linking the ethics of care with moral resilience; they identified only 6 eligible studies, none of which examined the relationship between the 2 concepts directly, leading them to conclude that empirical work in this area remains scarce. Fowler [16] revisited the history of nursing ethics and showed that the profession has long rested on a relational ethical foundation consistent with the core of the ethics of care, although this body of knowledge has not been systematically consolidated. The closest prior work is the qualitative systematic review by Waterfield and Barnason [17], which applied the ethics of care and Tronto’s [10] elements of care to nursing but focused on nurses’ workload in acute care settings and on the nurse-manager relationship rather than on synthesizing the components and indicators of the ethics of care as a whole.

Similarly, more recent systematic reviews of nursing ethics have tended to emphasize negative experiences, such as moral distress and organizational ethical climate [18] or nurses’ moral resilience [19]. Studies applying Tronto’s [10] framework directly to nursing have typically been confined to a single component or a single context; for example, 1 study of end-of-life care in a nursing home analyzed all 5 of Tronto’s [10] phases but remained limited to that 1 setting [20]. The resulting picture is fragmented across contexts and perspectives, and no review has yet consolidated and synthesized how each component of the ethics of care in the nursing profession is expressed through indicators or behaviors under a unified Gilligan [8] and Tronto [10] framework. Consistent with this, a search of the PROSPERO registry using the terms “ethics of care” and “care ethics” (searched on May 29, 2026) identified broadly related projects but none, other than the present review, aimed directly at synthesizing the components and indicators of the ethics of care in the nursing profession. This review therefore seeks to address that gap.

Accordingly, this qualitative systematic review aims to identify, appraise, and synthesize the components and indicators of the ethics of care in the nursing profession, following the frameworks of Gilligan [8] and Tronto [10], without restriction on publication year. It addresses 4 research questions (RQs):

1. What are the components of the ethics of care in the nursing profession?
2. What indicators or behaviors reflect each component?
3. Are there indicators or aspects, not yet captured by the frameworks of Gilligan [8] and Tronto [10], that emerge from the evidence?
4. How do the components and indicators vary across contexts?

The anticipated outcome is a clear set of definitions and behavioral indicators of the ethics of care in the nursing profession that can inform the subsequent development of assessment instruments, further research, and educational practice.

## Methods

### Type of Review and Synthesis Approach

This protocol describes a qualitative systematic review that will synthesize evidence using best-fit framework synthesis (BFFS), following the approach described by Carroll et al [21,22]. The review aims to identify, appraise, and synthesize evidence on the components and indicators of the ethics of care in nursing within an a priori framework comprising Gilligan’s [8] ethics of care as an overarching lens and Tronto’s [10] 5 elements of care as an operational structure. BFFS allows the existing evidence to be tested against the a priori framework and, where the evidence does not fit, allows that framework to be extended through inductive analysis. The review will be reported in accordance with the PRISMA (Preferred Reporting Items for Systematic Reviews and Meta-Analyses) 2020 guidelines, and the search will be reported in accordance with PRISMA-S (Preferred Reporting Items for Systematic Reviews and Meta-Analyses literature search extension) [23]; no meta-analysis will be conducted.

### Study Registration

This review protocol has been registered with PROSPERO (CRD420251032024). The registration record was created on April 13, 2025, and is publicly available on the PROSPERO website. The protocol was developed in accordance with the PRISMA guidelines [24]. Ethics approval was waived, as no data will be collected directly from human participants. The findings will be disseminated through publication in a peer-reviewed academic journal.

### Protocol Amendments

Several amendments were made to the registered protocol before completion of the review. First, the review approach was changed from a systematic review with meta-analysis to a qualitative systematic review using BFFS because the data of interest are conceptual and behavioral rather than quantitative effect estimates. Second, the original date restriction (2020-2024) was removed so that earlier and foundational work relevant to the ethics of care would not be excluded. Third, the database set was revised according to institutional access and methodological suitability: Scopus was removed because institutional access was unavailable, EBSCO Discovery Service was removed because it is a discovery layer rather than a discrete database, and PubMed (MEDLINE) was added. Backward citation searching of the reference lists of included studies was also added. Fourth, the language restriction was limited to English-language publications. Fifth, owing to resource constraints, screening and quality appraisal were planned primarily with a single primary reviewer, with calibration, maintenance of an audit trail, and consultation with a second reviewer in cases of uncertainty used as safeguards.

An initial search had been performed under the originally registered protocol (a systematic review with meta-analysis, with the registered database set and a 2020-2024 date restriction). Following the first round of peer review, the RQs, review approach, search strategy, database set, and eligibility criteria were substantially revised. Because these revisions materially changed the search, the initial search was not carried forward; the search was rerun in full under the amended approach in May 2026, and all records reported in this manuscript were identified through this new search. The records retrieved under the original protocol were therefore superseded and are not included in the counts reported in this manuscript. These amendments are reported here for transparency; the PROSPERO registration will be updated to reflect them following the current round of revision.

### RQs and Key Definitions

The review is structured according to the population, concept, and context framework, with the population comprising registered nurses and nursing students, the concept focusing on the ethics of care as conceptualized by Gilligan [8] and Tronto [10], and the context encompassing nursing practice and nursing education.

For clarity, the term “components” refers to the principal conceptual dimensions of the ethics of care, namely, Tronto’s [10] 5 elements, whereas “indicators” refers to the observable behaviors or expressions that reflect each component.

### Search Strategy

A systematic search was conducted across 5 electronic databases: CINAHL Complete (via EBSCOhost), PubMed (MEDLINE), ScienceDirect (Elsevier), SpringerLink (Springer Nature), and JSTOR. Two concept blocks were combined with the Boolean operator AND. The first block captures the concept of the ethics of care (“ethics of care” OR “care ethics” OR “ethical caring” OR “caring ethics” OR “relational ethics” OR “nursing ethics” OR “moral reasoning”), and the second captures the population (nurs* OR health OR health care OR “health care”). The inclusion of the terms health and health care in the population block was intended to increase the sensitivity of the search so as not to miss nursing-related work that may not use the term nurse explicitly; eligibility screening will then be restricted to work relevant to nursing.

Where supported, controlled vocabulary was applied (CINAHL headings in CINAHL and MeSH terms in PubMed [MEDLINE]), while ScienceDirect, SpringerLink, and JSTOR were searched using free-text terms. In ScienceDirect, full words were spelled out rather than truncated because the platform limits the number of Boolean operators and does not support truncation. Searches were limited to English-language documents, with no date restriction, and automatic term expansion was disabled. The database searches were supplemented by backward citation searching of the reference lists of included studies. All searches were conducted in May 2026. The full, line-by-line search strategy for each database is provided in Multimedia Appendix 1 [23], reported in accordance with PRISMA-S. During the search process in May 2026, a research librarian was consulted, who reviewed the search terms and methods and advised on the search approach.

### Eligibility Criteria

Studies will be included if they meet the following criteria: (1) population: registered nurses and nursing students, including interprofessional work only where data specific to nurses can be extracted separately; (2) concept: work that draws on Gilligan’s [8] or Tronto’s [10] ethics of care or that addresses any one of the framework’s components; (3) study design: qualitative, quantitative, and mixed methods research; (4) language and time frame: English-language publications, with no date restriction (from database inception to the search date); and (5) publication type: articles published in peer-reviewed journals (with the associated risk of publication bias acknowledged in the Discussion section).

Studies will be excluded if they are nonresearch documents, such as opinion pieces or editorials; do not address Gilligan’s [8] or Tronto’s [10] ethics of care in the nursing context; or are not available in full text for eligibility assessment. Here, “not available” refers to the inability to access the full text, not to incomplete information within an article. Quality appraisal using the JBI tools [25] will be used solely to inform the interpretation of findings and will not serve as a criterion for excluding studies.

### Study Selection (Screening)

References will be managed in EndNote 20 (Clarivate Analytics) and screened in Rayyan (Rayyan Systems Inc). Owing to resource constraints, screening will be carried out by a single primary reviewer. To reduce the risks associated with single-reviewer screening (such as missing relevant studies and selection bias), several safeguards will be applied: calibration with coreviewers on an initial sample of records, maintenance of an audit trail in Rayyan, and consultation with a second reviewer in cases of uncertainty. Single-reviewer screening will be acknowledged as a limitation in the Discussion section.

### Data Extraction

Data will be extracted using a standardized form that will first be piloted on a sample of included studies. The extracted data will include basic study information (title, authors, year, country, and setting), study details (design, objectives, population, and methods), and findings relevant to the review questions. In particular, data will be extracted on the components of the ethics of care addressed in each study, the conceptual definitions or descriptions of those components, the indicators or behavioral expressions reflecting each component, contextual factors related to their expression, and any additional indicators or aspects not fully captured by the frameworks of Gilligan [8] and Tronto [10]. For quantitative or mixed methods studies, narrative findings relevant to these components and indicators will also be extracted and mapped to the framework. The full line-by-line search strategies are provided in Multimedia Appendix 1, and the final data extraction form is provided in Multimedia Appendix 2.

### Quality Appraisal

The methodological quality of included studies will be appraised after inclusion using JBI’s critical appraisal tools appropriate to each study design, following the JBI Manual for Evidence Synthesis [25]. Depending on the design of the included studies, this may include, for example, the JBI Critical Appraisal Checklist for Qualitative Research or other relevant JBI tools. Each study will be assessed using the checklist appropriate to its design, and items rated as “unclear” or “not applicable” will be recorded separately. Owing to resource constraints, appraisal will be undertaken primarily by 1 reviewer, with discussion with a second reviewer in cases of uncertainty. Appraisal results will be used only to inform the interpretation of the findings and will not serve as a criterion for excluding studies; therefore, appraisal will not affect the counts in the PRISMA flow diagram.

### Data Synthesis

Data will be synthesized using the BFFS, following the approach described by Carroll et al [21,22]. Because the review proceeds within the a priori frameworks of Gilligan [8] and Tronto [10], BFFS allows the evidence to be tested against the framework and the framework to be extended from the evidence within a single process. The data to be synthesized are qualitative text extracted from included studies, such as findings, conceptual descriptions, and passages describing caring behavior. All analysis will be conducted in NVivo (version 15; **Lumivero**) in 6 steps.

1. The first step is constructing an a priori framework by defining initial codes (nodes) from Tronto’s [10] 5 elements (attentiveness, responsibility, competence, responsiveness, and reciprocity), with Gilligan’s [8] ethics of care guiding the overall interpretation
2. The second step is deductive coding, in which the extracted text is mapped onto the initial codes to address RQ1 and RQ2
3. The third step is inductive analysis, following the thematic synthesis approach of Thomas and Harden [26], for text that cannot be mapped onto any initial code, generating new themes or candidate nodes to identify indicators or aspects not captured by the existing framework, thereby addressing RQ3
4. The fourth step is examining the relationships among components and using matrix coding queries in NVivo to compare patterns across contexts within the profession (such as registered nurses, nursing students, care settings, and countries or cultures), thereby addressing RQ4
5. The fifth step is integrating the deductive and inductive findings into a revised best-fit framework that both confirms and extends the a priori framework
6. The sixth step is testing and refining the framework against the evidence over several iterations, so that the resulting framework is internally coherent and comprehensively accounts for the evidence

For included quantitative studies, findings will be interpreted narratively and mapped onto the relevant components and indicators; no statistical pooling or meta-analysis will be performed, as the review aims to synthesize qualitative definitions and indicators. The synthesis will be presented as a table of the characteristics of included studies; a table of synthesized indicators grouped under the 5 components, with operational definitions and frequency of occurrence; a diagram of the relationships among components and indicators; and an analysis of contextual variation.

## Results

This study received no external funding. The searches across the 5 databases were conducted in May 2026 and yielded 30,134 records; after the removal of 4855 duplicates, 25,279 records remained for screening. At the time of submission of this revised manuscript (May 2026), screening and data extraction were underway, and the review was anticipated to be completed by December 2026, with results expected to be published in 2027.

**Figure 2:**
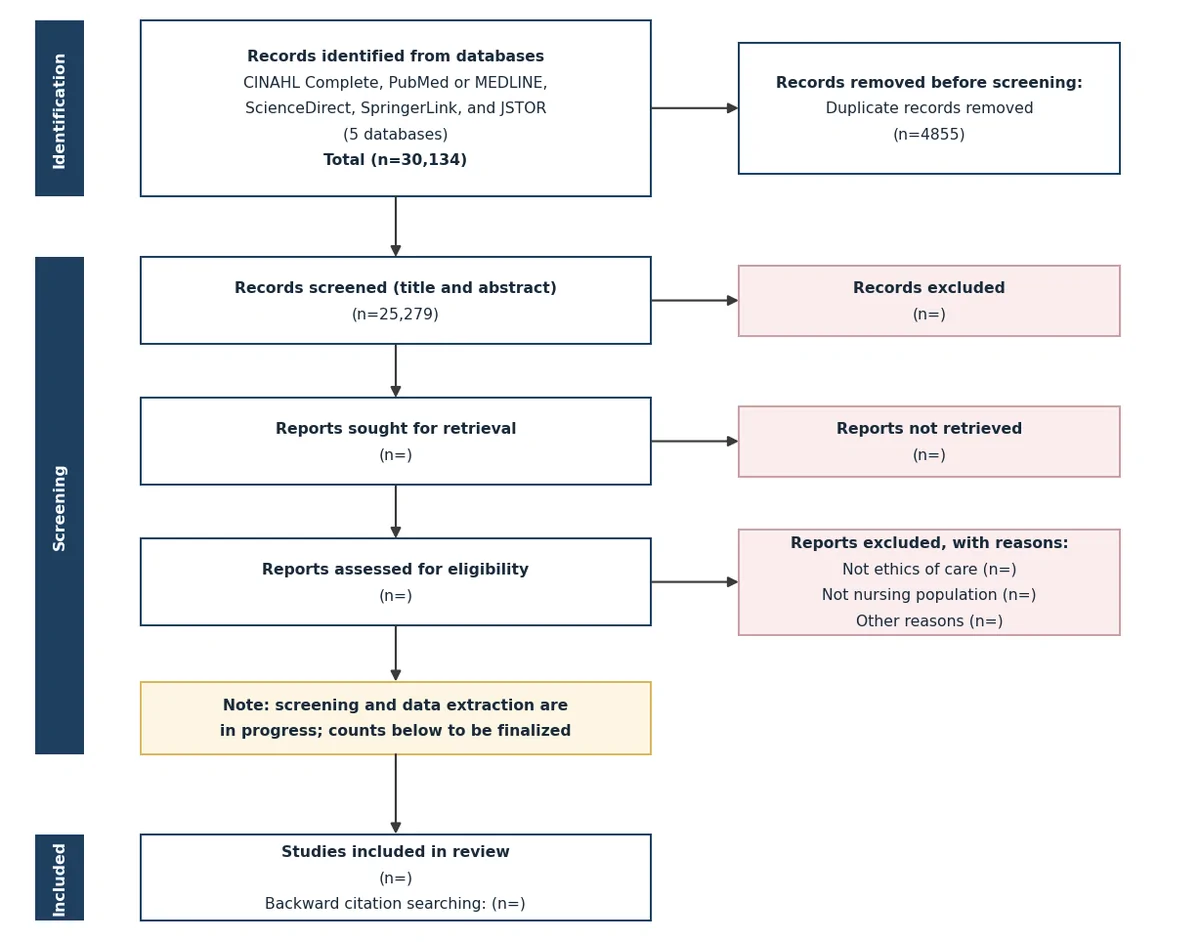
PRISMA (Preferred Reporting Items for Systematic Reviews and Meta-Analyses) 2020 flow diagram of study identification, screening, and selection for a qualitative systematic review of the components and behavioral indicators of the ethics of care among registered nurses and nursing students.

The PRISMA flow diagram (Figure 2) will present the number of records identified, the numbers excluded with reasons at each stage, and the final number of included studies, reported as a single, internally consistent set of figures. Amendments to the registered protocol are described in the Methods section.

## Discussion

### Principal Findings

On completion, the review is expected to yield clearer definitions and behavioral indicators for each of Tronto’s [10] 5 components, with Gilligan’s [8] ethics of care serving as an overarching lens, and to show how these indicators are expressed and how they differ when applied across various nursing contexts.

### Comparison With Prior Work

Most prior reviews have examined only particular aspects or components. Selvakumar and Kenny [15] reported that empirical work linking the ethics of care with moral resilience remains limited; Waterfield and Barnason [17] focused on nurses’ workload; and Klaver and Baart [27] examined a single component (attentiveness) and noted the absence of clear indicators for that aspect. Other reviews have tended to address negative experiences, such as moral distress and ethical climate [18] or moral resilience [19]. The present review differs by synthesizing the components and indicators of the ethics of care as a whole using the Gilligan [8] and Tronto [10] frameworks, which together encompass all relevant components. It is therefore expected to address a gap not addressed by previous reviews.

### Strengths and Limitations

A strength of this review is its systematic integration of the Gilligan [8] and Tronto [10] frameworks with BFFS, together with reporting in accordance with PRISMA 2020 and PRISMA-S, which renders the search and selection process transparent and auditable. Several limitations should be considered in interpreting the findings: screening by a single reviewer, albeit with bias-reduction safeguards; the restriction to peer-reviewed journal articles, which may introduce publication bias; differences in health systems and nursing roles across countries, which may affect the comparability of findings; the heterogeneity of definitions of the ethics of care across studies, which may complicate synthesis; and limitations in the database set due to institutional access, which may be expanded as access to additional databases becomes available. Backward citation searching was incorporated to help mitigate the risk of missing relevant studies.

Several amendments were made to the registered protocol to improve conceptual alignment and methodological feasibility. These changes, particularly the removal of the original date restriction and the adoption of the BFFS, may broaden the range of included studies and increase heterogeneity, but they are expected to improve the conceptual coherence of the synthesis and the relevance of the review to its RQs.

### Future Directions

The synthesized definitions and indicators can be extended in several directions: the development of evidence-based instruments for assessing the ethics of care, empirical studies to test the framework in real-world contexts, and nursing curricula designed to foster the ethics of care.

## Data Availability

The datasets extracted and synthesized during the review will be available from the corresponding author on reasonable request.

## Appendix

Multimedia Appendix 1 Search strings.

Multimedia Appendix 2 Data extraction form.

## Abbreviations

BFFS: best-fit framework synthesis
PRISMA: Preferred Reporting Items for Systematic Reviews and Meta-Analyses
PRISMA-S: Preferred Reporting Items for Systematic Reviews and Meta-Analyses literature search extension
RQ: research question

## References

[1] Fry ST, Johnstone MJ (2008). Ethics in Nursing Practice: A Guide to Ethical Decision Making, Third Edition.

[2] Kitson AL (1987). A comparative analysis of lay-caring and professional (nursing) caring relationships. Int J Nurs Stud.

[3] (2021). The ICN code of ethics for nurses. International Council of Nurses.

[4] Cheraghi R, Valizadeh L, Zamanzadeh V, Hassankhani H, Jafarzadeh A (2023). Clarification of ethical principle of the beneficence in nursing care: an integrative review. BMC Nurs.

[5] Dewi NM, Aryana IG, Bela IK, Widiastuti NK, Putra IK, Satriani NL (2024). Nursing ethics as a foundation in nursing practice: a literature review. Babali Nurs Res.

[6] Rest JR (1986). Moral Development: Advances in Research and Theory.

[7] Beauchamp T, Childress J (2019). Principles of biomedical ethics: marking its fortieth anniversary. Am J Bioeth.

[8] Gilligan C (1982). In A Different Voice: Psychological Theory and Women's Development.

[9] Tronto JC (1993). Moral Boundaries: A Political Argument for an Ethic of Care.

[10] Tronto JC (2013). Caring Democracy: Markets, Equality, and Justice.

[11] Keller J, Kittay EF, Garry A, Khader SJ, Stone A (2017). Feminist ethics of care. The Routledge Companion to Feminist Philosophy.

[12] Juujärvi S, Ronkainen K, Silvennoinen P (2019). The ethics of care and justice in primary nursing of older patients. Clin Ethics.

[13] Juujärvi S, Tetri B (2025). Care and justice reasoning in nurses' everyday ethics. Nurs Ethics.

[14] Jameton A (1984). Nursing Practice: The Ethical Issues.

[15] Selvakumar S, Kenny B (2021). Ethics of care and moral resilience in health care practice: a scoping review. Clin Ethics.

[16] Fowler M (2024). Nursing Ethics, 1880s to the Present: An Archaeology of Lost Wisdom and Identity.

[17] Waterfield D, Barnason S (2022). The integration of care ethics and nursing workload: a qualitative systematic review. J Nurs Manag.

[18] Xue K, Shang J, Yang C, Pan L, Shi H, Zeng Y (2025). Nurses' moral distress and ethical climate: a systematic review and meta-analysis. Nurs Ethics.

[19] Yang C, Xue K, Shang J, Pan L, Shi H, Jin Y, Zeng Y (2026). Moral resilience among nurses: a systematic review and meta-analysis. Nurs Ethics.

[20] Gilbert R, Lillekroken D (2024). Nurses' perceptions of how their professional autonomy influences the moral dimension of end-of-life care to nursing home residents: a qualitative study. BMC Nurs.

[21] Carroll C, Booth A, Cooper K (2011). A worked example of "best fit" framework synthesis: a systematic review of views concerning the taking of some potential chemopreventive agents. BMC Med Res Methodol.

[22] Carroll C, Booth A, Leaviss J, Rick J (2013). "Best fit" framework synthesis: refining the method. BMC Med Res Methodol.

[23] Rethlefsen ML, Kirtley S, Waffenschmidt S, Ayala AP, Moher D, Page MJ, Koffel JB (2021). PRISMA-S: an extension to the PRISMA statement for reporting literature searches in systematic reviews. Syst Rev.

[24] Page MJ, McKenzie JE, Bossuyt PM, Boutron I, Hoffmann TC, Mulrow CD, Shamseer L, Tetzlaff JM, Akl EA, Brennan SE, Chou R, Glanville J, Grimshaw JM, Hróbjartsson A, Lalu MM, Li T, Loder EW, Mayo-Wilson E, McDonald S, McGuinness LA, Stewart LA, Thomas J, Tricco AC, Welch VA, Whiting P, Moher D (2021). The PRISMA 2020 statement: an updated guideline for reporting systematic reviews. BMJ.

[25] Aromataris E, Munn Z (2020). JBI Manual for Evidence Synthesis.

[26] Thomas J, Harden A (2008). Methods for the thematic synthesis of qualitative research in systematic reviews. BMC Med Res Methodol.

[27] Klaver K, Baart A (2011). Attentiveness in care: towards a theoretical framework. Nurs Ethics.

