# Supplementary material for: Ethics of Care in Nursing: Protocol for a Qualitative Systematic Review and Best-Fit Framework Synthesis
Source: JMIR Res Protoc. 2026 Sep 10;15:e85172. doi: 10.2196/85172 (PMC13613045; doi:10.2196/85172)
Supplement: Supplementary file 1 [file resprot_v15i1e85172_app1.docx]

**Multimedia Appendix 1. Full Electronic Search Strategies (PRISMA-S)**

*Manuscript #85172: Ethics of Care in Nursing: Protocol for a Qualitative Systematic Review and Best-Fit Framework Synthesis*

Searches conducted: May 2026. Language limit: English. Date limit: none (database inception to search date). This appendix is reported following the PRISMA-S checklist (Rethlefsen et al., 2021).

**1. Overview of information sources**

Five electronic bibliographic databases were searched, supplemented by backward citation searching (checking the reference lists of all included studies). Two concept blocks were combined with the Boolean operator AND: Block 1 (ethics-of-care concept) and Block 2 (population). Within each block, terms were combined with OR. Controlled vocabulary (CINAHL Headings; MeSH) was applied where supported; free-text searching was used elsewhere. Phrases were enclosed in double quotation marks to search as exact phrases. Automatic term expansion / “apply equivalent subjects” was turned off.

**Concept blocks (all databases)**

| **Block** | **Search terms (combined with OR)** |
| --- | --- |
| Block 1 Ethics of care | "ethics of care" OR "care ethics" OR "ethical caring" OR "caring ethics" OR "relational ethics" OR "nursing ethics" OR "moral reasoning" |
| Block 2 Population | nurs* OR health OR healthcare OR "health care" |
| Combination | Block 1 AND Block 2 |

*Note. In ScienceDirect, truncation (*) and a large number of Boolean operators are not supported; therefore, full words were spelled out (e.g., nurse OR nurses OR nursing) and the strategy was simplified accordingly (see 2.4).*

**2. Database-specific search strategies**

**2.1 CINAHL Complete (EBSCOhost)**

Fields: title (TI) and abstract (AB) plus CINAHL Headings (MH). Limiters: English language. Apply equivalent subjects: off.

```
S1  (MH "Ethics, Nursing")
S2  TI ( "ethics of care" OR "care ethics" OR "ethical caring" OR
        "caring ethics" OR "relational ethics" OR "nursing ethics"
        OR "moral reasoning" )
    OR AB ( "ethics of care" OR "care ethics" OR "ethical caring" OR
        "caring ethics" OR "relational ethics" OR "nursing ethics"
        OR "moral reasoning" )
S3  S1 OR S2
S4  (MH "Nurses+") OR (MH "Nursing Practice+")
S5  TI ( nurs* OR health OR healthcare OR "health care" )
    OR AB ( nurs* OR health OR healthcare OR "health care" )
S6  S4 OR S5
S7  S3 AND S6
Limiters: English Language
```

*Subject headings marked (MH …) to be confirmed against the current CINAHL Headings thesaurus by the searcher.*

**2.2 PubMed (MEDLINE)**

Fields: title/abstract [tiab] plus MeSH terms [Mesh]. Filter: English[lang].

```
("ethics of care"[tiab] OR "care ethics"[tiab] OR
 "ethical caring"[tiab] OR "caring ethics"[tiab] OR
 "relational ethics"[tiab] OR "nursing ethics"[tiab] OR
 "moral reasoning"[tiab] OR "Ethics, Nursing"[Mesh])
AND
(nurs*[tiab] OR health[tiab] OR healthcare[tiab] OR
 "health care"[tiab] OR "Nurses"[Mesh])
Filters: English[lang]
```

*MeSH terms to be confirmed in the MeSH browser by the searcher.*

**2.3 SpringerLink (Springer Nature)**

Free-text search. Phrases in quotation marks. SpringerLink does not support truncation, so full word forms were entered. English; no date limit.

```
("ethics of care" OR "care ethics" OR "ethical caring" OR
 "caring ethics" OR "relational ethics" OR "nursing ethics" OR
 "moral reasoning")
AND
(nurse OR nurses OR nursing OR health OR healthcare OR "health care")
```

**2.4 ScienceDirect (Elsevier)**

Free-text search. No truncation supported; limited Boolean operators. Full words spelled out. English; no date limit. (Owing to the platform’s operator limit, the strategy was run in parts and the results combined.)

```
("ethics of care" OR "care ethics" OR "nursing ethics" OR
 "moral reasoning")
AND
(nurse OR nurses OR nursing OR health OR healthcare)
```

**2.5 JSTOR**

Free-text search. Phrases in quotation marks. English; no date limit.

```
("ethics of care" OR "care ethics" OR "ethical caring" OR
 "caring ethics" OR "relational ethics" OR "nursing ethics" OR
 "moral reasoning")
AND
(nurs* OR health OR healthcare OR "health care")
```

**2.6 Supplementary search**

Backward citation searching: the reference lists of all studies included after full-text screening were checked to identify additional relevant studies not retrieved by the database searches.

**3. Summary of records retrieved**

| **Stage** | **Records** |
| --- | --- |
| Records identified across the five databases | 30,134 |
| Duplicates removed | 4,855 |
| **Records after de-duplication (entered screening)** | **25,279** |

Records were imported into EndNote and de-duplicated; screening was performed in Rayyan.

Note. The five databases yielded 30,134 records in total; after the removal of 4,855 duplicates, 25,279 records remained and entered the screening stage. These figures correspond to those reported in the PRISMA flow diagram (Figure 1) and in the Results and Abstract of the manuscript.

**Reference**

Rethlefsen ML, Kirtley S, Waffenschmidt S, et al. PRISMA-S: an extension to the PRISMA Statement for reporting literature searches in systematic reviews. Syst Rev. 2021;10:39. doi:10.1186/s13643-020-01542-z
