# Supplementary material for: Ethics of Care in Nursing: Protocol for a Qualitative Systematic Review and Best-Fit Framework Synthesis
Source: JMIR Res Protoc. 2026 Sep 10;15:e85172. doi: 10.2196/85172 (PMC13613045; doi:10.2196/85172)
Supplement: Supplementary file 2 [file resprot_v15i1e85172_app2.docx]

**Multimedia Appendix 2. Data Extraction Form**

*Ethics of Care in Nursing: Protocol for a Qualitative Systematic Review and Best-Fit Framework Synthesis*

One form is completed per included study. The form is piloted on a sample of included studies and refined before full extraction. Fields map to the four research questions (RQ1–RQ4). Quantitative and mixed-methods studies: extract narrative findings relevant to the components and indicators and map them to the framework.

## A. Identification

| **Study ID** | *e.g., 001* |
| --- | --- |
| **Reviewer (initials)** |  |
| **Date of extraction** |  |

## B. Bibliographic information

| **First author (et al.)** |  |
| --- | --- |
| **Year of publication** |  |
| **Full title** |  |
| **Country** |  |
| **Journal / source** |  |
| **Language** |  |

## C. Study characteristics

| **Study design** | *qualitative / quantitative / mixed-methods* |
| --- | --- |
| **Aim / objectives** |  |
| **Population** | *registered nurses / nursing students / mixed — specify* |
| **Sample size & characteristics** |  |
| **Data collection method** |  |
| **Data analysis method** |  |
| **Setting / clinical context** |  |

## D. Framework-mapped findings (RQ1 – components; RQ2 – indicators)

For each of Tronto’s five elements, record how the study defines or describes the component and the observable indicators or behaviours it reports. Leave blank if the component is not addressed.

| **Component (Tronto)** | **Conceptual definition / description as used in the study (RQ1)** | **Indicators / observable behaviours (RQ2)** | **Supporting text or quotation (location / page)** |
| --- | --- | --- | --- |
| **Attentiveness** |  |  |  |
| **Responsibility** |  |  |  |
| **Competence** |  |  |  |
| **Responsiveness** |  |  |  |
| **Reciprocity** |  |  |  |

## E. Emergent indicators / aspects (RQ3)

Content that does not map onto any a priori component and may extend the framework.

| **Indicator / aspect NOT captured by Gilligan or Tronto** | **Description and rationale** | **Supporting text (location)** |
| --- | --- | --- |
|  |  |  |
|  |  |  |
|  |  |  |

## F. Contextual variation (RQ4)

| **Contextual dimension** | **Detail from study** | **Note on how indicators vary** |
| --- | --- | --- |
| Professional / educational stage (nursing student vs registered nurse) |  |  |
| Clinical setting |  |  |
| Country / culture |  |  |
| Other contextual factor |  |  |

## G. Quality appraisal (JBI)

| **JBI checklist used** | *specify tool appropriate to the study design* |
| --- | --- |
| **Criteria met / total** |  |
| **Items rated “unclear” or “not applicable”** |  |
| **Overall methodological quality (notes)** |  |

*Note: Appraisal informs the interpretation of findings only; it is not used as a criterion for excluding studies and does not affect the PRISMA flow counts.*

## H. Reviewer notes

| **Uncertainty requiring second-reviewer consultation?** | *Yes / No* |
| --- | --- |
| **Decision / resolution** |  |
| **Additional notes** |  |
